# International medical electives for medical students at a German university: a secondary analysis of longitudinal data

**DOI:** 10.1093/inthealth/ihab009

**Published:** 2021-03-03

**Authors:** Ralf Weigel, Lara Wiegand, Stefanie Balzereit, Michael Galatsch

**Affiliations:** Witten/Herdecke University, Faculty of Health, School of Medicine, Alfred-Herrhausen Street 50, 58448 Witten, Germany; Friede Springer endowed professorship for global child health, Witten/Herdecke University, Faculty of Health, School of Medicine, Alfred-Herrhausen Street 50, 58448 Witten, Germany; Witten/Herdecke University, Faculty of Health, School of Medicine, Alfred-Herrhausen Street 50, 58448 Witten, Germany; Witten/Herdecke University, Faculty of Health, School of Medicine, Alfred-Herrhausen Street 50, 58448 Witten, Germany; Witten/Herdecke University, Faculty of Health, School of Medicine, Alfred-Herrhausen Street 50, 58448 Witten, Germany; Friede Springer endowed professorship for global child health, Witten/Herdecke University, Faculty of Health, School of Medicine, Alfred-Herrhausen Street 50, 58448 Witten, Germany

**Keywords:** global health, medical education, medical students

## Abstract

**Background:**

International medical electives (IMEs) are entry points to global health opportunities. IME uptake at German universities is unclear. We analyse 14 y of IME.

**Methods:**

Student registry data were collected. Univariate linear regression examined relationships between enrolment year and IMEs.

**Results:**

The median (IQR) number of IMEs of all enrolment years was 54 (32–80) and 51 (38–67)% of all students took an IME. Enrolment year significantly predicted IME frequency and the proportion of students taking IMEs.

**Conclusions:**

Student interest in IMEs is increasing. Universities should invest more broadly in IME opportunities for student, faculty and university enrichment.

## Introduction

International medical electives (IMEs) are often the first exposure of medical students to global health. They can be a compulsory part of global or public health courses or organised as electives by students themselves. An IME may last from several weeks to several months. Students value the international experience to enhance their clinical skills and to aid understanding of different health systems.^1^ Thus, universities in the UK, Australia and North America offer IME guidelines and support before, during and after these experiences.^2–5^

In line with the German government's internationalisation and new global health agendas, universities are striving to become more internationally focused on research and education. However, educational activities in global health at undergraduate and postgraduate level are underdeveloped.^6^ In particular, knowledge about the extent of IMEs is lacking. Thus, this study aims to analyse the frequency and uptake of IMEs over 14 y at a single university, Witten/Herdecke University (UW/H).

## Methods

Until April 2008, the UW/H enrolled one cohort of students annually; from October 2008, UW/H enrolled two cohorts per year. Each cohort had 42 students. From year 2, all students take compulsory clinical electives, including IMEs in years 4 and 5. Students are solely responsible for the preparation, communication and logistics of an IME. Students identify and contact the foreign institution, find a local supervisor, provide IME details to the UW/H subject chair (mostly professors) and inform the registry at UW/H. The registry keeps paper forms and letters. Selected information about students’ electives is also entered into an MS Excel spreadsheet, version 16 (Microsoft corporation, Redmond, US), for tracking and reporting purposes.

Excel table data from June 2003 to May 2019 included 2187 electives of 548 students; 1546 of the electives (70.7%) were categorised as IMEs. Summary statistics were only calculated for the 1122 (51.3%) IMEs of student intakes that completed 5 y of study before the end of the data collection in May 2019. Univariate linear regression examined the relationship between calendar year of enrolment (independent variable), number of IMEs and the proportion of students with IMEs (dependent variables) using SPSS version 25 (IBM, Amonk, US).

## Results

**Figure 1.**
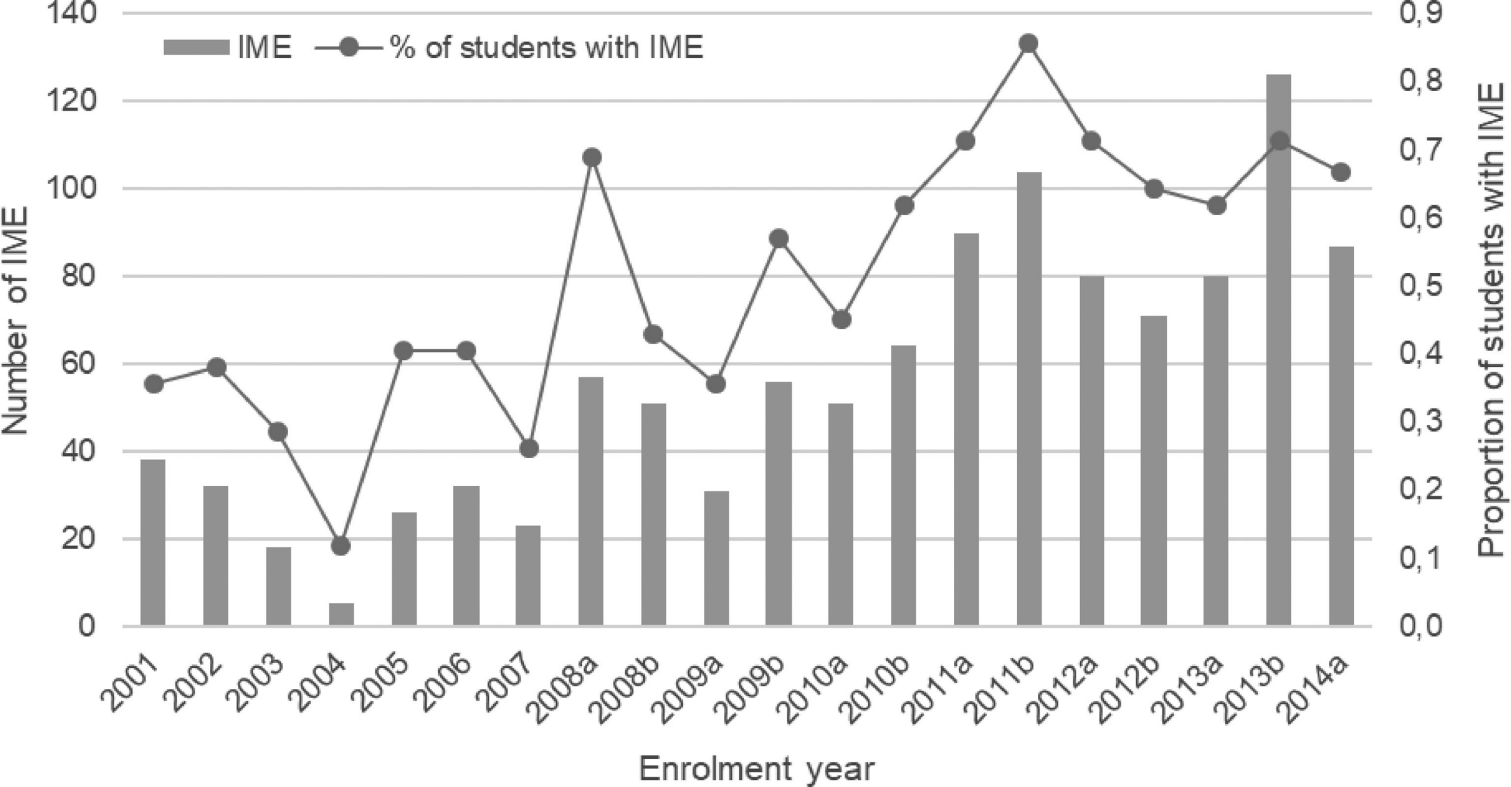
International medical electives (IMEs) completed by medical students at Witten/Herdecke University, Germany. The IMEs of students enrolled from 2015 to 2019 were excluded because those students had not completed their studies by the end of data collection.

From 2001 to 2014, 431 students took 1122 IMEs while at university (Figure 1), excluding IMEs of students enrolled from 2015 to 2019 as they had not completed their 5 y of study. The median (IQR) number of IME recorded per enrolment year across this period was 54 (32–80) and 51 (38–67)% of students had at least one IME while at university. In regression analysis, the enrolment year significantly predicted the number of IMEs (β=0.858, p<0.001) and the proportion of students with IMEs (β=0.782, p<0.001), explaining 72.1% and 58.9% of the variance, respectively.

## Discussion

Over 14 y, the frequency of IMEs and the proportion of students taking IMEs significantly increased with calendar year of enrolment. These findings suggest increased IME uptake in line with the government's promotion of internationalisation and global health. Heightened interest in IMEs informs the future practice of IME monitoring and utilisation.

IME monitoring is not routine. Illustration of the extent of IMEs among medical students required dedicated data collection, cleaning and analysis at the university level. The lack of systematic data collection and a reporting system is a barrier and a missed opportunity to use findings for decision-making at individual faculties and to inform national policies. Routine, prospective data collection of students’ international exposures could help optimisation of the IME experience. For example, it would allow students interested in IMEs to draw on previous students’ experiences by easily accessing their peers’ reports and contacting them. Further, faculty could identify common IME locations and develop closer bonds with hosts, not only for teaching, but also for collaborative research. Quality data would also improve the chances for successful applications to donors interested in funding IMEs or broader international health exchanges.

The reported increase in IMEs among students also highlights the need for more structured preparation of IMEs that go beyond individual student effort. In other developed countries, IME initiatives are often identified, organised or otherwise supported by the university or an individual faculty and are not reliant on the students themselves. The opposite appears to be true at medical faculties in Germany, where the IME burden falls upon students. A structured approach facilitated by the university, department or faculty, including a combination of predeparture and post-return workshops, could maximise the benefits of IMEs for students^4^ and allow bilateral exchange programmes. Additional support may also free students to better reflect on their expectations for skills acquisition during their IMEs, identification of their own knowledge limitations and to better prepare for the experience in a culturally sensitive manner.

## Conclusions

This study demonstrates increased IME frequency and uptake over time, setting an example for other universities in Germany. Improved monitoring and reporting of IMEs will document growing interest in IMEs at medical faculties and support additional IME investment across student, faculty, university and national stakeholders.

## Data Availability

Data are available upon request from the corresponding author.
